# Gender Differences for the Prevalence and Risk Factors of Workplace Violence Among Healthcare Professionals in Shandong, China

**DOI:** 10.3389/fpubh.2022.873936

**Published:** 2022-05-02

**Authors:** Long Sun, Wen Zhang, Fei Qi, Yani Wang

**Affiliations:** ^1^Centre for Health Management and Policy Research, School of Public Health, Cheeloo College of Medicine, Shandong University, Jinan, China; ^2^National Health Commission of China (NHC) Key Lab of Health Economics and Policy Research, Shandong University, Jinan, China; ^3^Binzhou People Hospital, Binzhou, China; ^4^Qingdao Municipal Center for Disease Control and Prevention, Qingdao, China

**Keywords:** workplace violence, prevalence, risk factor, gender difference, healthcare professionals, China

## Abstract

**Background:**

Workplace violence (WPV) against healthcare professionals (HPs) has been recognized as important occupational health and societal problem in the world. Many studies were also conducted to explore the prevalence, risk factors, and adverse outcomes of WPV against HPs. Although the gender differences in the prevalence and risk factors of WPV against HPs have been implied in many studies, fewer studies were conducted to explore the gender differences for WPV against HPs, especially in China. In this study, we aim to analyze the gender differences in the prevalence and risk factors of WPV against HPs in Shandong, China.

**Methods:**

This study was conducted among HPs with a cross-sectional design. WPV, social-demographic variables, occupational characteristics, physical disease, social support, and depression were evaluated for the participated HPs. The prevalence and risk factors of WPV among male healthcare professionals (MHPs) and female healthcare professionals (FHPs) were analyzed in this study. Student's *t*-tests, one-way ANOVA, and logistic regressions were performed to test the associated factors of WPV among MHPs and FHPs.

**Results:**

The prevalence of WPV among MHPs and FHPs was 61.4 and 48.8%, respectively. Being silent was the most common method of response to WPV among MHPs (52.3%) and FHPs (59.2%). For MHPs, the associated factors of WPV were master's degree (odds ratio (OR) =2.20, *P* < 0.05), bachelor's degree (OR = 2.49, *P* < 0.001), lower income level (OR = 1.81, *P* < 0.05), manager (OR = 1.81, *P* < 0.05), and depression (OR = 1.05, *P* < 0.001). For FHPs, the associated factors of WPV were a master's degree (OR = 1.58, *P* < 0.05), more working hours per week (OR = 1.02, *P* < 0.001), and depression (OR = 1.05, *P* < 0.001).

**Conclusion:**

The prevalence of WPV among MHPs was higher than FHPs, and the associated factors for WPV against HPs were also different among MHPs and FHPs. The findings remind us that some gender-specific interventions are needed to control WPV against HPs.

## Background

Workplace violence (WPV) is defined as “incidents where staff is abused, threatened, or assaulted in circumstances related to their work” according to WHO (1). Because of the close contact with the patients and their relatives for healthcare professionals (HPs), HPs are at high risk of WPV, which also has been recognized as important occupational health and societal problem in the world (2–4). As we know, HPs are the dominating health services providers, and they play very important roles in the quality and outcomes of health services. Therefore, WPV against HPs should be paid attention to the world.

In recent decades, many studies about WPV toward HPs were performed around the world, and we also achieved several major findings on WPV toward HPs. The first achievement was about the high prevalence of WPV against HPs. A recent meta-analysis study with a total of 331,544 participants reported that the prevalence of WPV against healthcare workers was 61.9% (5). Some other review studies reported a wide range of the prevalence of WPV (9.5%−97.6%) among different kinds of HPs in different regions (6–8). The second achievement was about the adverse outcomes of WPV on HPs. Until now, several adverse outcomes of WPV to HPs had been identified, such as physical and mental health problems (9, 10), poor quality of life (11), poor sleep quality (12), and negative work-related outcomes (13). The next achievement was about the associated factors for WPV against HPs. Several social-demographic (14, 15) and work-related characteristics (16–19) were also identified to be associated with WPV among HPs.

We should know that all of these previous publications gave us important information about WPV against HPs. When we reviewed these publications about WPV worldwide, we could easily find gender differences in the prevalence of WPV against HPs. However, the results were conflicting. Some studies supported the higher prevalence of WPV among male healthcare professionals (MHPs) (20–22), and some studies supported the higher prevalence of WPV among female healthcare professionals (FHPs) (23). Some studies reported there were no differences between MHPs and FHPs (24). As we know, the occupational classifications of medical professionals in Chinese hospitals, like many other hospitals in the world, are highly segregated by gender. For example, there are more females among nurses, and there are more males among surgeons. The association between gender discrimination and WPV among HPs was also supported in the previous study (25). On the other side, gender differences are also one of the characteristics of Confucianism in China. For men, they mainly undertake the economic responsibility in their family, and they may be influenced by the work-related factors. For women, they mainly take care of their families, and they may be influenced by their family events. The differences may result from the different outcomes of WPV between MHPs and FHPs. All of these remind us that there should be some gender differences for WPV toward HPs in China.

Actually, there were also some studies, which explored the gender differences in WPV against HPs. However, most findings in these studies were broad-brush, and the detailed information was less explored in these studies. When we reviewed these publications, we could find that the main findings in these studies were also about the high prevalence of WPV among MHPs (26, 27). In another study, it found the gender differences in WPV reporting and the experiences of different kinds of WPV (28). The gender differences in their responses and risk factors of WPV were less reported in previous studies.

To explore the gender differences in WPV against HPs, we conducted a cross-sectional study among HPs in general hospitals in Shandong province, China. We hypothesize the higher prevalence of WPV among MHPs than FHPs, and more MHPs may respond to WPV by counterattack than FHPs. The factors associated with WPV among MHPs and FHPs may be also different. In this study, we want to explore the gender differences in the prevalence, the responses, and the risk factors of WPV against HPs. The findings can give us important implications for the policies and interventions of WPV against HPs. If the gender differences for WPV against HPs were built, it also implies to us that some gender-specific interventions are needed to control WPV against HPs.

## Methods

### Study Sample and Design

This study was conducted among Chinese HPs worked in general public hospitals in Shandong province, China. For Shandong province, its population ranked second (29), and the number of healthcare workers ranked first in all the Chinese provinces (30). In this study, a cross-sectional design with multiple stratified random cluster sampling was used to recruit the HPs in general public hospitals. First, we randomly selected three municipalities from all the 17 municipalities in Shandong province. Second, in each of the selected municipalities, three counties/districts were randomly selected. Third, one municipal hospital was randomly selected from each of the municipalities, and one county-level/district-level hospital was selected from these counties or districts. Totally, three municipal hospitals and nine county-level/district-level hospitals were selected to conduct the survey in this study. In the municipal hospitals, we selected three inpatient areas from each department. In the county-level/district-level hospitals, we selected two inpatient areas from each department. The inclusion criteria of the HPs were the ones who had signed the labor contracts with the selected hospitals. HPs who were receiving training in the selected hospitals were excluded from this study. Finally, there were 3,426 valid questionnaires, which were analyzed in this study. The valid response rate was 88.9% (3,426/3,852).

### Data Collection

The survey was performed between December 2018 and January 2019. Two trained postgraduate students were asked to be in the hospitals, and the hospital managers helped them to dispense the questionnaires to the HPs. The HPs were asked to fill out the questionnaires anonymously. The two postgraduate students were in the hospital to answer the questions about the study and questionnaires. There were not any rewards for the HPs.

### Measures

#### Workplace Violence and HPs' Response

Workplace violence (WPV) was evaluated by the self-reported question that “have you ever experienced the following behavior conducted by your patients or their relations?” The answers could be chosen from verbal violence (VV), physical violence (PV), both physical and verbal violence (BPV), and none. A similar question was also used and evaluated to measure WPV in previous studies (31, 32). In this study, the prevalence of WPV was calculated by the whole sample. As the main aim of this study was to analyze the gender differences for WPV among MHPs and FHPs, we analyzed the factors associated with WPV among MHPs and FHPs, respectively.

#### Social-Demographic Variables

Age was calculated by the HPs' date of birth. Marital status was assessed by single, married, divorced, widowed, and others. As there were few responses about the last 3 answers, married status was recoded into single, married, and others. Education was evaluated by the academic degree, that the HPs received. As most of them received bachelor's degrees and above, we recorded them into degrees, master's degrees, bachelor's degrees, and others.

#### Occupational Characteristics

Types of HPs included doctors, nurses, and medical technicians. The professional title was evaluated by senior, vice-senior, intermediate, junior, and others. Income level was evaluated by the question about the HPs' total income per month. The answers were ≤ 3,000 RMB, 3,001–5,000 RMB, 5,001–7,000 RMB, 7,001–9,000 RMB, 9,001–11,000 RMB, 11,001–13,000 RMB, and ≥13,001 RMB (7 RMB ≈ 1 dollar). We recoded it into ≤ 5,000 RMB (L1), 5,001–9,000 RMB (L2), and ≥9,001 RMB (L3). The manager was interviewed with the question “do you have a management position?” The answers were yes and no. As all the surveyed hospitals were municipal or county-level hospitals, hospital level was assessed by level 2 and level 3. Working hours per week were measured by the self-reported averaged working hours per week, and we analyzed the numbers of the working hours per week. Years of working were evaluated by the self-reported years of working for the HPs.

#### Physical Disease

The physical disease was assessed by the question “have you been diagnosed with any physical diseases?” The answer was yes and no.

#### Social Support

Social support was evaluated by the Multidimensional Scale of Perceived Social Support (MSPSS) (33, 34), and its Chinese version had been tested and used with nice validity and reliability in previous studies (35). On this scale, there are 12 items with seven answers from 1 (strongly disagree) to 7 (strongly agree). The sum of these items was calculated and analyzed in this study, and the higher scores mean a higher level of social support. In this study, the Cronbach's alpha of MSPSS was 0.958, and the Guttman split-half coefficient of MSPSS was 0.936.

#### Depression

The Center for Epidemiologic Studies-Depression Scale (CES-D) was used to measure the level of depression (36), and its Chinese version was also tested with good reliability and validity (37, 38). The CES-D scale contains 20 items to evaluate the frequency of subjects' depressive symptoms in the last week, and the responses were from 0 (<1 day) to 3 (5–7 days). The higher sum of these items replaces a higher level of depressive symptoms, which was analyzed in this study. In this study, the Cronbach's alpha of CES-D was 0.852, and the Guttman split-half coefficient of CES-D was 0.854.

### Statistical Analysis

Data analyses were performed by the IBM SPSS Statistics 24.0 (Web Edition). Shapiro–Wilk test was used to test the normality of the data. Student's *t*-tests or chi-square tests were used to compare the differences between MHPs and FHPs. The method of Wilson score was used to calculate the 95% CI for the prevalence of WPV, PV, and VV without continuity correction (39). Binary logistic regressions with the entering method were performed to test the associated factors of WPV among MHPs and FHPs. WPV was analyzed as the dependent variable, and all other factors were analyzed as the independent variables. All of the tests were two-tailed and a *p*-value of ≤ 0.05 was considered statistically significant.

## Results

In this cross-sectional study, we interviewed 3,426 HPs worked in general public hospitals. Among these HPs, most of them were females (2,507/3,426, 73.2%). The detailed social-demographic and occupational information for these HPs was shown in the second column in Table 1. We also compared the differences in these factors between MHPs and FHPs. We found that MHPs had older age (*t* = 9.61, *P* <0.001), higher academic degree (χ^2^ = 118.72, *P* < 0.001), more doctors (χ^2^= 890.95, *P* < 0.001), higher professional title (χ^2^= 137.02, *P* < 0.001), higher income level (χ^2^ = 74.25, *P* < 0.001), more managers (χ^2^ = 41.98, *P* < 0.001), more physical disease (χ^2^= 15.23, *P* < 0.001), more working hours (*t* = 12.22, *P* < 0.001), more years of working (*t* = 3.10, *P* < 0.01), lower social support (*t* = −4.53, *P* < 0.01), and higher level of depression (*t* = 2.91, *P* < 0.01) than FHPs. The detailed results were shown in the last 3 columns in Table 1.

**Table 1:** Sample description and single analyses for gender differences in the sample [*n* (%)].

**Variables**	**All**	**MHPs**	**FHPs**	***t/*χ^2^**
Observations	3,426 (100.0)	919 (26.8)	2,507 (73.2)	–
Age (years)	35.14 ± 8.42	37.40 ± 9.61	34.32 ± 7.78	9.61***
Married status				0.28
Single	577 (16.8)	155 (16.9)	422 (16.8)	
Married	2,802 (81.8)	753 (81.9)	2,049 (81.8)	
Others	47 (1.4)	11 (1.2)	36 (1.4)	
Education				118.72***
Doctor degree	56 (1.6)	23 (2.5)	33 (1.3)	
Master degree	562 (16.4)	250 (27.2)	312 (12.4)	
Bachelor degree	2,368 (69.2)	559 (60.8)	1,809 (72.2)	
Others	440 (12.8)	87 (9.5)	353 (14.1)	
Types of HPs				890.95***
Doctors	1,268 (37.0)	660 (71.8)	608 (24.3)	
Nurses	1,695 (49.5)	73 (8.0)	1,622 (64.7)	
Medical technicians	463 (13.5)	186 (20.2)	277 (11.0)	
Professional title				137.02***
Senior	109 (3.2)	60 (6.5)	49 (2.0)	
Vice-senior	303 (8.8)	139 (15.1)	164 (6.5)	
Intermediate	1,170 (34.2)	337 (36.7)	833 (33.2)	
Junior and others	1,844 (53.8)	383 (41.7)	1,461 (58.3)	
Income level				74.25***
L1	1,615 (47.1)	337 (36.7)	1,278 (51.0)	
L2	1,571 (45.9)	477 (51.9)	1,094 (43.6)	
L3	240 (7.0)	105 (11.4)	135 (5.4)	
Manager				41.98***
Yes	659 (19.2)	243 (26.4)	416 (16.6)	
No	2,767 (80.8)	676 (73.6)	2,091 (83.4)	
Hospital level				2.14
Level 3	1,477 (43.1)	415 (45.2)	1,062 (42.4)	
Level 2	1,949 (56.9)	504 (54.8)	1,445 (57.6)	
Physical disease				15.23***
Yes	457 (13.3)	157 (17.1)	300 (12.0)	
No	2,969 (86.7)	762 (82.9)	2,207 (88.0)	
Working hours/week	47.69 ± 9.27	50.89 ± 11.33	46.52 ± 8.41	12.22***
Years of working	10.98 ± 8.91	11.77 ± 10.03	10.69 ± 8.45	3.10**
Social support	62.46 ± 13.82	60.69 ± 14.64	63.10 ± 13.45	−4.53**
Coping skill	30.63 ± 9.83	29.92 ± 10.15	30.88 ± 9.69	−2.55*
Depression	14.72 ± 10.38	15.57 ± 11.15	14.40 ± 10.07	2.91**

***p < 0.001; **p < 0.01; *p < 0.05. HPs, healthcare professionals; MHPs, male healthcare professionals; FHPs, female healthcare professionals. L1 denotes to ≤ 5,000 RMB monthly income. L2 denotes to 5,001–9,000 RMB monthly income. L3 denotes to ≥9,001 RMB monthly income.

In Table 2, we analyzed the prevalence of WPV in this sample. The results showed that the prevalence of WPV, PV, VV, and BPV was 47.8% (95% CI 46.1–49.5%), 1.3% (95% CI 1.0–1.7%), 37.9% (95% CI 36.3–39.6%), and 13.0% (95% CI 11.9–14.2%), respectively. Among MHPs, the prevalence of WPV, PV, VV, and BPV was 61.4% (95% CI 58.2–64.5%), 1.8% (95% CI 1.2–2.9%), 37.4% (95% CI 34.4–40.6%), and 22.1% (95% CI 19.5–24.9%), respectively. For FHPs, the prevalence of WPV, PV, VV, and BPV was 48.8% (95% CI 46.9–50.8%), 1.1% (95% CI 0.7–1.6%), 38.1% (95% CI 36.2–40.0%), and 9.7% (95% CI 8.6–10.9%), respectively. We also found that the prevalence of WPV among MHPs were higher than them among FHPs (χ^2^ = 42.43, *P* < 0.001).

**Table 2:** Prevalence of different types of workplace violence among male and female healthcare professionals [% (95% CI)].

	**All**	**MHPs**	**FHPs**	**χ^2^**
Observations	3,426	919	2,507	
WPV	47.8 (46.1–49.5)	61.4 (58.2–64.2)	48.8 (46.9–50.8)	42.43***
PV	1.3 (1.0–1.7)	1.8 (1.2–2.9)	1.1 (0.7–1.6)	105.42***
VV	37.9 (36.3–39.6)	37.4 (34.4–40.6)	38.1 (36.2–40.0)	
BPV	13.0 (11.9–14.2)	22.1 (19.5–24.9)	9.7 (8.6–10.9)	
None	47.8 (46.1–49.5)	38.7 (35.5–41.8)	51.1 (49.2–53.1)	

***p < 0.001; WPV, workplace violence; PV, physical violence; VV, verbal violence; BPV, both PV and VV; MHPs, male healthcare professionals; FHPs, female healthcare professionals.

We also analyzed the different responses to WPV among MHPs and FHPs experienced different kinds of WPV in Table 3. We could find that most HPs chose to be silent as the method of response to WPV (57.0%). For MHPs, the descending rankings of responses to WPV were silence (52.3%), call police (17.6%), others (13.1%), counterattack (11.9%), and no response (5.1%). The same ranking was also found for FHPs experienced different kinds of WPV. The silence was also the most common method of response to PV, VV, and BPV for MHPs and FHPs. We also found that the responses to WPV (χ^2^ = 9.70, *P* < 0.05) were statistically different between MHPs and FHPs experienced WPV, and the gender differences for PV, VV, and BPV were not supported in our results (all *p* > 0.05).

**Table 3:** Response to different kinds of WPV among MHPs and FHPs experienced workplace violence [*n* (%)].

**Responses**	**All**	**MHPs**	**FHPs**	**χ^2^**
WPV	1,788 (100.0)	564 (31.5)	1,224 (68.5)	9.70*
Counterattack	171 (9.6)	67 (11.9)	104 (8.5)	
Silence	1,020 (57.0)	295 (52.3)	725 (59.2)	
Calling police	296 (16.6)	99 (17.6)	197 (16.1)	
No response	91 (5.1)	29 (5.1)	62 (5.1)	
Others	210 (11.7)	74 (13.1)	136 (11.1)	
PV	1,299 (100.0)	344 (26.5)	955 (73.5)	5.05
Counterattack	121 (9.3)	41 (11.9)	80 (8.4)	
Silence	783 (60.3)	496 (57.0)	587 (61.4)	
Calling police	161 (12.4)	40 (11.6)	121 (12.7)	
No response	68 (5.2)	19 (5.5)	49 (5.1)	
Others	166 (12.8)	48 (14.0)	118 (12.4)	
VV	44 (100.0)	17 (38.6)	27 (61.4)	3.19
Counterattack	2 (4.5)	1 (5.9)	1 (3.7)	
Silence	26 (59.1)	11 (64.7)	15 (55.6)	
Calling police	12 (27.3)	3 (17.6)	9 (33.3)	
No response	1 (2.3)	0 (0.0)	1 (3.7)	
Others	3 (6.8)	2 (11.8)	1 (3.7)	
BPV	445 (100.0)	203 (45.6)	242 (54.4)	5.76
Counterattack	45 (10.1)	24 (11.8)	21 (8.7)	
Silence	219 (49.2)	89 (43.9)	130 (53.7)	
Calling police	117 (26.3)	56 (27.6)	61 (25.2)	
No response	22 (5.0)	10 (4.9)	12 (5.0)	
Others	42 (9.4)	24 (11.8)	18 (7.4)	

*p < 0.05. WPV, workplace violence; PV, physical violence; VV, verbal violence; BPV, both PV and VV; MHPs, male healthcare professionals; FHPs, female healthcare professionals.

Binary logistic regressions were further conducted to analyze the factors associated with WPV among MHPs and FHPs. The results showed that WPV were associated with master degree [odds ratio (OR) = 2.20, *P* < 0.05, Ref. = others], bachelor degree (OR = 2.49, *P* < 0.001, Ref. = others), L2 income level (OR = 1.92, *P* < 0.05, Ref. = L3 income level), manager (OR = 1.81, *P* < 0.05), and depression (OR = 1.05, *P* < 0.001) among MHPs. Among FHPs, WPV were associated with master degree (OR = 1.58, *P* < 0.05, Ref. = others), working hours (OR = 1.02, *P* < 0.001), and depression (OR = 1.05, *P* < 0.001). The detailed information was shown in Table 4.

**Table 4:** Logistic regressions for the factors associated with workplace violence, among MHPs and FHPs [OR (95% CI)].

**Variables**	**MHPs**	**FHPs**
Observations	919	2,507
Age	1.02 (0.99, 1.05)	1.01 (0.98, 1.03)
Married status (Ref. = Others)		
Single	2.46 (0.54, 11.34)	1.69 (0.81, 3.54)
Married	3.44 (0.80, 14.89)	1.31 (0.65, 2.64)
Education (Ref. = Others)		
Doctor degree	2.44 (0.77, 7.75)	1.76 (0.75, 4.12)
Master degree	2.20 (1.17, 4.16)*	1.58 (1.06, 2.35)*
Bachelor degree	2.49 (1.46, 4.25)***	1.26 (0.98, 1.63)
Types of medical staff (Ref. = Medical technicians)		
Doctors	1.28 (0.86, 1.91)	1.12 (0.81, 1.55)
Nurses	0.65 (0.34, 1.25)	1.00 (0.76, 1.33)
Professional title (Ref. = Junior and others)		
Senior	0.90 (0.34, 2.35)	0.86 (0.40, 1.83)
Vice-senior	0.81 (0.41, 1.59)	0.64 (0.40, 1.04)
Intermediate	1.13 (0.74, 1.72)	1.08 (0.84, 1.38)
Income level (Ref.= L3)		
L1	1.27 (0.68, 2.35)	0.95 (0.61, 1.49)
L2	1.92 (1.01, 3.34)*	1.13 (0.74, 1.72)
Manager (Ref. = No)	1.81 (1.14, 2.86)*	1.18 (0.90, 1.56)
Level 3 Hospital (Ref. = Level 2 Hospital)	0.80 (0.58, 1.13)	0.93 (0.77, 1.12)
Physical disease (Ref. = No)	1.23 (0.79, 1.92)	1.07 (0.82, 1.40)
Working hours/week	1.01 (1.00, 1.03)	1.02 (1.00, 1.03)***
Years of working	0.99 (0.96, 1.02)	1.02 (1.00, 1.03)
Social support	1.01 (1.00, 1.02)	1.00 (0.99, 1.00)
Depression	1.05 (1.03, 1.06)***	1.05 (1.04, 1.06)***
Constant	0.01***	0.09***
R^2^	0.17	0.11***

***p < 0.001; *p < 0.05. MHPs, male healthcare professionals; FHPs, female healthcare professionals; OR, odds ratio; CI, confidence interval. L1 denotes to ≤ 5,000 RMB monthly income. L2 denotes to 5,001–9,000 RMB monthly income. L3 denotes to ≥ 9,001 RMB monthly income.

## Discussion

There were several critical findings on the gender differences of WPV against HPs in this study. The first one was the higher prevalence of WPV against HPs among MHPs. We also found that most HPs chose silence as the response to WPV, PV, VV, and BPV. The gender difference for the responses to WPV was supported, and it was not statistically significant for PV, VV, and BPV. Several factors associated with WPV among MHPs and FHPs were also found in this study, such as education and depression. For MHPs, lower income levels and managers were at a higher risk of WPV, and more working hours were associated with a higher risk of WPV among FHPs.

The first finding of the higher prevalence of WPV among MHPs was not new. As we mentioned above, previous studies also supported the higher prevalence of WPV among MHPs in China (40, 41). However, there were some studies, which reported a higher prevalence of WPV among FHPs in Western countries (23, 24, 42). The reason may be explained by the Chinese traditional culture about the weak position of women in their workplaces, and they are relatively less to be treated as targets of WPV. In this study, we also reported a lower prevalence of WPV (47.8%), compared with previous findings (about 60%) in China (41). It may be caused by the recent reforms in the Chinese healthcare system and medical education, which was discussed in the previous study (43).

The other finding in this study was about the responses to WPV among HPs experienced WPV, and we found that silence was the most common response to WPV among HPs (>50%), especially for FHPs (59.2%). The results were similar to other studies in the world (44–46). One of the reasons may be that most of the WPV against HPs are not very serious to HPs, and the HPs do not need to take action the response. The other reason may be that being silent may be one of the best choices to control further harm from WPV. For FHPs, silence may be a better choice because of their weak status in physical strength.

We also found that both education and depression were positively associated with WPV among MHPs and FHPs. Actually, both of the factors had been identified to be associated with WPV in many previous studies (47–49). The association between education and WPV may be caused by the differences in the patients they served. HPs with higher education may serve more serious patients, and these patients are also at higher risk of negative outcomes of health. These negative outcomes of healthcare services may result in WPV from these patients' relatives. Actually, the positive association between depression and WPV had been identified in previous studies (50). As we know, WPV against HPs is a kind of violence or threats to HPs, and both violence and threat were risk factors for depression, which may also cause depressive symptoms (48).

One of the main aims of this study was to analyze gender differences for the factors associated with WPV. For MHPs, we found that lower income levels and managers were at higher risk of WPV. These two factors were also supported to be associated with WPV among different kinds of HPs (51, 52). As we know, in Chinese families and traditional culture, men should take more responsibility in their families than women, and this makes them may care about their income. In this situation, a lower income level may result from a higher level of job burnout (53), which is also a risk factor for WPV (54, 55). For male managers, they need to deal with more problems about WPV than females in their hospitals or departments because of the culture of female protection, which makes them at higher risk of WPV.

For FHPs, we found that more working hours were associated with a higher risk of WPV. In previous studies, the association between age and WPV was conflicting (14, 26, 56). In this study, the positive association was supported by our sample. One of the reasons may be caused by the time frame of WPV. In this study, the time frame of WPV was a lifetime, which makes age positively associated with WPV. For the positive association between working hours and WPV among FHPs, it may be caused by the work-family conflict for females. For most of the females, they need to take care of their families, and long working hours may result in work-family conflict (57), which may further result in higher work stress and WPV (58, 59).

Based on the previous discussion, we can easily assume the different associated factors of WPV between MHPs and FHPs. For MHPs, WPV was mainly associated with income and career development. For FHPs, WPV was mainly associated with family-related factors. Actually, similar findings were also supported in previous studies among general practitioners in China (52). The differences can be explained by the Chinese traditional culture—“males master outside, females master inside.” It means that men take responsibility for the economic and social status in the family, and women take responsibility for family work.

In this study, we have several critical findings on the gender differences for WPV. However, there were also some limitations, which may also bring some bias to the findings. First, because of the cross-sectional design, we cannot get any causal relationships for the relationship between these factors and WPV. Second, the survey was conducted among HPs working in Chinese level 2 and 3 public hospitals, and the findings may be not be suitable for other kinds of HPs working in other regions and level 1 hospitals. Third, WPV and the related factors were collected by the HPs' self-reporting in this study, which may also bring some bias to the results. Fourth, the time frame of WPV was a lifetime, and the prevalence of WPV may be higher than in other studies with a shorter time frame.

In this study, we analyzed the gender differences in prevalence and risk factors of WPV among HPs in Chinese general hospitals. The results supported the higher prevalence of WPV among MHPs, and silence was the most common method of response to WPV, especially for FHPs. For MHPs, the associated factors of WPV were education, depression, lower income level, and manager. For FHPs, the associated factors of WPV were education, depression, older age, and more working hours. The findings imply to us that there are gender differences for WPV among HPs, and some gender-specific interventions are needed to control WPV against HPs.

## Data Availability Statement

The raw data supporting the conclusions of this article will be made available by the authors, without undue reservation.

## Ethics Statement

The studies involving human participants were reviewed and approved by Institutional Review Board of Shandong University School of Public Health (ref.: 20181219). The patients/participants provided their written informed consent to participate in this study.

## Author Contributions

LS analyzed the data and drafted the manuscript. WZ collected the data and commented on the draft of this manuscript. FQ and YW designed the study. All authors read and approved the final manuscript.

## Funding

The research was supported by the National Natural Science Foundation of China (71974114).

## Conflict of Interest

The authors declare that the research was conducted in the absence of any commercial or financial relationships that could be construed as a potential conflict of interest.

## Publisher's Note

All claims expressed in this article are solely those of the authors and do not necessarily represent those of their affiliated organizations, or those of the publisher, the editors and the reviewers. Any product that may be evaluated in this article, or claim that may be made by its manufacturer, is not guaranteed or endorsed by the publisher.
